# Both sides now: modeling motor regulation of microtubule length at both ends

**DOI:** 10.1088/1478-3975/ae600a

**Published:** 2026-04-24

**Authors:** Maria-Veronica Ciocanel, Bhargav R Karamched

**Affiliations:** 1 Department of Mathematics, Duke University, Durham, NC, United States of America; 2 Department of Biology, Duke University, Durham, NC, United States of America; 3 Department of Mathematics, Florida State University, Tallahassee, FL, United States of America; 4 Institute of Molecular Biophysics, Florida State University, Tallahassee, FL, United States of America; 5 Program in Neuroscience, Florida State University, Tallahassee, FL, United States of America

**Keywords:** microtubule dynamics, molecular motors, partial differential equations, emergent length regulation

## Abstract

Microtubules are dynamic biopolymers whose lengths are continuously regulated by the concerted actions of polymerization, depolymerization, and motor-protein activity. While numerous mathematical models have explored the regulation of filament length, most have been formulated in the context of growth and shrinking at a single tip of a microtubule, effectively ignoring the mechanistic description of complex phenomena such as treadmilling. Here, we develop a multiscale model for microtubule length regulation that explicitly couples the kinetics of two classes of kinesin molecular motors to filament dynamics at both microtubule tips. Motor densities along the filament are modeled using one-dimensional parabolic partial differential equations. The microtubule length evolves dynamically through a shrinkage term that depends on motor density and which closes the system. In the adiabatic regime, where motor kinetics are fast relative to length dynamics, we derive a reduced model amenable to analytic study and identify simple parameter relationships distinguishing growth, disassembly, and treadmilling behavior. Numerical simulations of the full system reveal qualitatively distinct dynamical regimes and demonstrate how bidirectional motor transport modulates filament length distributions. We parametrize our model with both *in vivo* and *in vitro* data and thus lay the foundation for developing mathematical models yielding a better understanding of cytoskeleton dynamics in living cells.

## 1. Introduction

Understanding the mechanisms that regulate the dimensions of subcellular compartments to match cell volume and metabolic needs remains a fundamental challenge in cell biology. Current evidence suggests that organelle dimensions are governed by a combination of physical limitations and autonomous self-organization [1]. Researchers have broadly categorized these regulatory strategies into several main types, notably quantal synthesis, molecular rulers, and dynamic balance [2].

The principle of dynamic balance dictates that the continuous turnover of structural components can yield a stable size, provided that the rates of building and dismantling the structure eventually equalize. Crucially, if either of these kinetic rates is intrinsically tied to the structure’s current dimensions, the system naturally contracts to a steady-state size. This is exemplified by microtubules in eukaryotic flagella, which experience ongoing turnover at their distal ends. A uniform breakdown rate is counteracted by an elongation rate that scales with length—a consequence of a finite pool of motor proteins delivering building blocks from the soma. This equilibrium ultimately dictates the final length of the flagellum [3–5].

Similar equilibrium models have been proposed for actin-rich protrusions like inner ear stereocilia [6]. In these systems, actin filaments undergo continuous treadmilling: they are degraded at their proximal base while growing at their distal tips. Because the addition of new material relies on the passive diffusion of actin monomers to the far end, the growth rate inherently decreases as the structure lengthens. A comparable diffusion-limited growth model has also been hypothesized to govern hook length in bacterial flagella [7].

Conversely, yeast cells utilize a distinct balancing strategy to manage microtubule length. In this case, specific kinesins travel processively to the ends of microtubules to actively promote depolymerization. Because longer microtubules offer a larger surface area to capture these depolymerizing kinesins from the surrounding cytosol, the rate of structural breakdown increases directly with length. Pairing this length-dependent degradation with a constant rate of polymerization ensures the microtubule reaches a definitive steady-state size [8–13]. Other related models of microtubule regulation focus on adjusting their dynamic balance, specifically through the targeted control of catastrophe frequencies [14, 15].

Here, we develop a multiscale model that focuses on the dynamic balance of microtubules. Microtubules are directionally-polarized filaments with biophysically distinguishable (+) and (–) ends [16]. The principal functions of microtubules are twofold: (1) maintain structural integrity of cell shape, and (2) aid in transport of vesicles and organelles throughout the interior of a cell. They also play a key role in cell motility during mitosis [16, 17].

There are two broad categories of microtubules: centrosomal and non-centrosomal. Centrosomal microtubules emerge from the pericentriolar material surrounding the centrioles of the centrosome. Their (–) ends are anchored to the centrosome and are thus not subject to dynamic growth and shrinking. The (+) ends extend out radially in the cytoplasm. This type of microtubule is prominent in most dividing animal cells. Non-centrosomal microtubules emanate from other microtubule organizing centers, such as the Golgi apparatus, nuclear envelope, or even from other microtubules via branching nucleation or severing. Both (+) and (–) ends are free and subject to growth and shrinking. They are prominent in polarized cells, such as neurons and other specialized cells, and support local organization rather than global radial structure [18, 19]. In this work, we focus on non-centrosomal microtubules, as they exhibit a variety of dynamical behaviors, such as growth, shrinking, and treadmilling.

Transport and delivery of vesicles and organelles is carried out by molecular motors, such as kinesin and dynein. Although the precise mechanism by which these motors collectively transport vesicles is debated, it is known that the polarity at a given end of the microtubule dictates what kind of molecular motor will travel along the microtubule in a given direction. For example, kinesin motors generally walk in the (+) direction along microtubules, whereas dynein motors tend to walk in the (–) direction. The transport and delivery process enabled by molecular motors is crucial for cellular and organism health. Breakdown in these processes in neurons has been implicated in neurodegenerative diseases such as Alzheimer’s and Huntington’s [20–22].

The question of how microtubule length is controlled therefore reflects an interesting balance between distinct effects. On the one hand, microtubules are continuously switching between states of growth and shrinking to facilitate cytoskeleton rebuilding and robustness to injury; on the other hand, they are required to maintain a semblance of constant length to facilitate robust vesicle transport throughout a cell. Understanding the mechanisms that generate specific microtubule length distributions is therefore of interest to both theorists and experimentalists.

Situations like this are precisely where mathematical models can play a key role in advancing biological understanding—namely to theorize outcomes for hypothesized mechanisms underlying a particular biological phenomenon. Indeed, a plethora of mathematical models exist that investigate some aspect of microtubule length control. However, these models tend to focus on the dynamics at one end of centrosomal microtubules (the (+) end) and are typically framed in the context of *in vitro* experiments. Several biological and mathematical models have been specifically proposed to address an important growth control mechanism for microtubules, where filament assembly is limited by length- or age-driven microtubule disassembly [23, 24]. While an exact molecular basis for this length-dependent catastrophe mechanism has not been fully established, experiments have supported several hypotheses. One is the ‘antenna’ mechanism, where certain types of depolymerizing kinesin motors accumulate at microtubule (+) ends and promote catastrophe [9, 25]. Another is a mechanism that proposes that, as microtubules grow, their tips become more ragged due to the variable protofilament lengths and are thus destabilized [26, 27].

**Figure 1.**
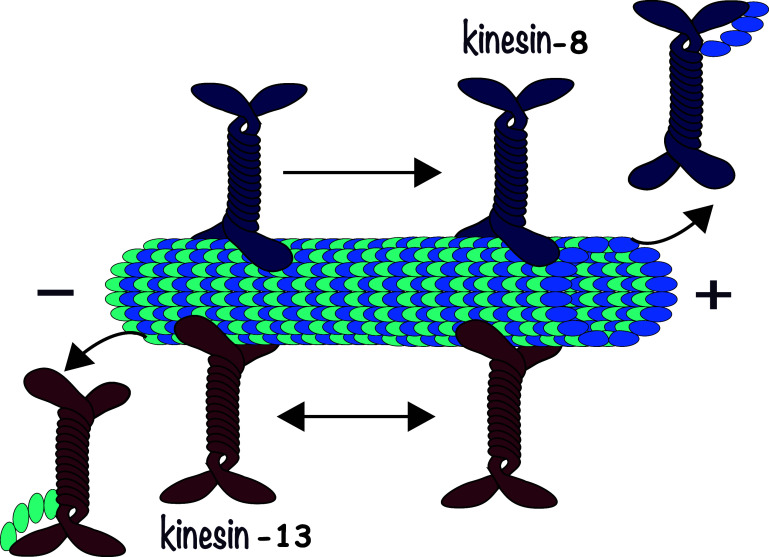
Schematic showing the bidirectional motor-based depolymerization of microtubules. These dynamics provide the foundation of our theoretical model.

Here, we develop a model of microtubule length dynamics at both ends, with parameters informed by *in vivo* data, coupled with a model of molecular motor kinetics, parametrized using *in vitro* measurements. We assume a length regulation paradigm where shrinkage is dependent on the density of depolymerizing molecular motors attached to microtubules. We explicitly model the dynamics of two types of molecular motors in the kinesin family and link their kinetics to overall microtubule length. We use a 1D macroscopic parabolic partial differential equation to model kinesin motor density dynamics. Such an equation can be derived in a principled way from a more detailed biophysical model formulated in terms of a stochastic hybrid system where a particle switches according to a Markov process between states where it moves ballistically in the (+) direction, ballistically in the (–) direction, or remains stationary [28, 29]. The domain of the advection-diffusion equations describes the length of the microtubule and thus changes through time (figure 1).

Our approach is reminiscent of the earlier model [25], which proposed the first mathematical formulation for the antenna model of MT depolymerization due to motor accumulation. This model tracked the density of depolymerizing Kip3p (kinesin-8) motors along a microtubule and showed that different motor binding models could not be distinguished. As they were interested in different phases of MT depolymerization, this study did not model individual microtubule end dynamics and instead focused on reproducing the decrease in microtubule length due to the depolymerizing motors. In addition, our approach is most similar to the model in [15], which also describes the density of depolymerizing motors along a single microtubule. As in [25], they consider a single class of motors that depolymerizes microtubules from the (+) end, and do not distinguish between bound and unbound motor states. This study then couples the microtubule length to the density of motors at the last site on the filament. A more complex version of the model they propose includes dynamic instability at the (+) end and incorporates a catastrophe rate that varies with length. By modulating the catastrophe frequency as a function of the density or the flux of depolymerizing motors, [15] makes analytical approximations of the mean MT length in these two theoretical scenarios. However, this study does not intend to model the dynamics of non-centrosomal microtubules, which undergo growth and shrinking at both filament ends, and does not distinguish between the contribution of depolymerizing motors and other mechanisms to the length-dependent catastrophe of microtubules.

Our model therefore goes beyond these prior studies by incorporating dynamics at (+) and (–) ends of microtubules, informing parameters from *in vivo* and *in vitro* data whenever possible, and assessing the contribution of motor-driven and non-motor-driven length-dependent disassembly. Exploring the parameter space of this model allows us to investigate the qualitative differences of the system’s behavior in various regimes. In the adiabatic regime, where motor dynamics are assumed to occur faster than microtubule length dynamics, we derive a reduced model that is amenable to analytic techniques. Using homotopy continuation, we analyze equilibria of the reduced model and describe regions of parameter space where different types of microtubule behavior (e.g. growth, disassembly, treadmilling) can be observed. Specifically, our reduced model provides a simple relationship between model parameters to distinguish between growth and disassembly in the adiabatic regime.

In the unrestricted parameter regime, we perform numerical simulations of the model on a growing domain and are also able to identify qualitatively distinct microtubule behaviors in various parameter regions. We are thus able to assess the impact of motor kinetics and different length-dependent disassembly mechanisms on microtubule dynamics. The main contribution of our work is thus the development of a flexible model that can shed light on the regimes of biophysical parameters that characterize microtubule length regulation. Such quantitative tools are especially useful given that ascertaining characteristics of *in vivo* behavior of microtubules remains challenging.

## 2. Background on microtubule-depolymerizing motors

As mentioned above, one of the ways in which microtubules achieve length regulation is through a length-dependent catastrophe mechanism, which has been observed both *in vitro* and *in vivo* [24]. Microtubules have been found to experience increased catastrophe frequency as they grow, and a leading mechanism for this phenomenon is the so-called ‘antenna’ mechanism, where kinesin motor proteins walk to microtubule ends and stimulate catastrophe. Studies on the kinesin-8 motor (Kip3P in the budding yeast) found that more motors accumulate at the (+) ends of longer microtubules, which in turn leads to further destabilization of these ends [9, 25]. Kinesin-8 was also found to be a slow, highly processive motor in [9], leading to the analogy of the microtubule filament to an antenna that directs Kip3p molecules to its (+) end and thus leads to a concentration gradient of the motor along the microtubule. These findings hold *in vitro* and *in vivo* and have positioned kinesin-8 as a length-dependent depolymerase that controls microtubule length from the (+) ends [9].

On the other hand, kinesin-13 motor proteins (MCAK and Klp10A) were found to depolymerize microtubules from both ends, though depolymerization was faster from the (–) ends [9, 30]. Unlike kinesin-8, kinesin-13 is known to start depolymerization by bending the terminal subunits of microtubules, leading to their loss of lateral interactions [25, 30]. The MCAK kinesin-13 protein is known to diffuse in a 1D random walk along the microtubule filament, as opposed to using directed motion like kinesin-8 [25, 31]. These studies have thus established kinesin-13 as a depolymerase that uses rapid diffusion to get to both ends of the microtubule, and which is able to cover short distances rapidly [31]. The protein Patronin was found to help (–) ends resist to complete depolymerization by kinesin-13, and the regulation of dynamic instability by these motors was thus found to be responsible for controlling mitotic spindle length *in vivo* [30].

## 3. Model of motor kinetics and microtubule length dynamics

Motivated by the biological evidence outlined in section 2, we develop a model that describes the motion of both (+) and (–)-end depolymerizing motors along microtubules. Neurons are long structures and, since we are interested in the dynamics of microtubules along such structures, we model space as one-dimensional, implicitly assuming all significant dynamics occur along the length of the neuron. Our goal is to model the impact of (+) and (–)-end motors on microtubule length. This setting yields the following coupled system of partial differential equations:

\begin{align*} \frac{\partial p}{\mathrm{d}t} &amp; = -v_+ \frac{\partial p}{\mathrm{d}x} + D_+ \frac{\partial^2 p}{\mathrm{d}x^2} + k_{\mathrm{on},+} c - k_{\mathrm{off},+}p\,,\end{align*}

\begin{align*} \frac{\partial c}{\mathrm{d}t} &amp; = D \frac{\partial^2 c}{\mathrm{d}x^2} - k_{\mathrm{on},+} c + k_{\mathrm{off},+}p \,,\end{align*}

\begin{align*} \frac{\partial m}{\mathrm{d}t} &amp; = v_- \frac{\partial m}{\mathrm{d}x} + D_- \frac{\partial^2 m}{\mathrm{d}x^2} + k_{\mathrm{on},-} f - k_{\mathrm{off},-}m\,,\end{align*}

\begin{align*} \frac{\partial f}{\mathrm{d}t} &amp; = D \frac{\partial^2 f^-}{\mathrm{d}x^2} - k_{\mathrm{on},-} f + k_{\mathrm{off},-}m \,,\end{align*}

where $p(x,t)$ is the density of MT-bound (moving) (+)-end motors, $c(x,t)$ is the density of unbound (+)-end motors, $m(x,t)$ is the density of MT-bound (moving) (–)-end motors, and $f(x,t)$ is the density of unbound (–)-end motors. The motors move with speeds $v_{+/-}$ and diffuse with diffusion coefficients $D_{+/-}$ along microtubules. Motors diffuse with diffusion coefficient *D* when unbound from microtubules. The binding rates of the motors to microtubules are $k_{\mathrm{on},+/-}$ and their unbinding rates are given by $k_{\mathrm{off},+/-}$. We denote the MT length by *L*, which extends from $x = L_-$ to $x = L_+$.

The advection-diffusion equations in equations (1) and (3) are effective mean-field reduction equations of a more detailed stochastic biophysical model describing the transport of a population of motors of a given type, which randomly switch between a motile state moving ballistically in the (+) direction, a motile state moving ballistically in the (–) direction, and a stationary state. Assuming the transitions between states are fast relative to the transport dynamics, one can employ a quasi-steady state approximation upon the associated Chapman–Kolmogoroff equations to obtain an effective partial differential equation for the dynamics. The diffusivity describes the modification to motor-cargo dynamics arising from the intermittent stationary state [28, 32].

We need to specify boundary conditions at the (+) and (–) ends for both the motor densities and for the MT ends. At the (–) end, we model a constant source of moving and diffusing (+)-end motors, as well as of diffusing (–)-end motors:

\begin{align*} p\left(L_-\right) &amp; = 1\,, \; c\left(L_-\right) = 1\,, \; \frac{\partial m}{\partial x} |_{L_-} = 0\,,\; f\left(L_-\right) = 1\,,\end{align*}

while at the (+) end, we model a constant source of moving and diffusing (–)-end motors and of diffusing (+) end motors as follows:

\begin{align*} \frac{\partial p}{\partial x}|_{L_+} &amp; = 0\,, \; c\left(L_+\right) = 1\,,\; m\left(L_+\right) = 1\,, \; f\left(L_+\right) = 1\,.\end{align*}

The boundary conditions for the moving (+)-end motors at the right edge of the domain and for the moving (–)-end motors at the left end of the domain assert that the Fickian flux of motors departing the domain is zero. The justification for imputing zero Fickian flux upon the motors is again derived from the underlying stochastic model of the advection-diffusion equations. In the stochastic model, motion across the boundary of the domain occurs in the motile, ballistic states [28, 33]. Thus, we allow for convective flux but set Fickian flux to naught.

We assume that the dynamics of the (+) and (–) ends of the MTs is given by:

\begin{align*} \begin{split} \frac{\partial L}{\partial t}|_{L_-} &amp; = -\alpha_- +\beta_-|L|+\gamma m\\ \frac{\partial L}{\partial t}|_{L_+} &amp; = \alpha_+ - \beta_+|L| - \gamma p, \end{split}\end{align*}

where the first term corresponds to intrinsic growth of the MTs, the second term denotes intrinsic length-dependent MT disassembly, and the third term models MT shrinking driven by the (+) and (–) end motor populations, respectively. These equations do not explicitly model the switch from growth to shrinking of MTs, but rather the overall dynamics at the (+) and (–) ends. We therefore incorporate the length-dependent shrinking mechanism in the second term of these equations.

## 4. Tractable model simplifications

Equations (1) and (7) describe a model that can be derived from first principles, and solving them numerically provides insight into how solutions behave. However, they are analytically difficult to parse. We thus invoke some approximations to obtain analytic insights from the model. Specifically, we reduce the model in equations (1) and (7) into a coupled set of ODEs to explore the parameter space of the model and examine the biophysical insights that emerge.

### 4.1. Fast-switching limit

We assume that switching occurs on a faster timescale than the advective and diffusive processes so that $k_{\mathrm{on},\pm}$ and $k_{\mathrm{off},\pm}$ are larger than $v_+/L^*$ and $D_{\pm}/L^{*2}$, where $L^*$ is the equilibrium microtubule length attained in the absence of motor influence. This has been observed experimentally in the peripheral sensory neurons of *Drosophila* [34]. We make this explicit with the mapping $k_{\mathrm{on/off,\pm}} \to \varepsilon^{-1}k_{\mathrm{on/off},\pm}$, where $0 < \varepsilon \ll 1$. The effective dynamics then manifest as a perturbation away from the stationary measure of the process governing the switching dynamics. We show the derivation explicitly for (+) moving motors and the derivation for (–) moving motors is nearly identical.

We rewrite the dynamics of the (+) moving motors as

\begin{equation*} \frac{\partial}{\partial t}\mathbf{p} = \varepsilon^{-1}\mathbf{A}\mathbf{p} + \mathbb{L}\left(\mathbf{p}\right),\end{equation*}

where

\begin{equation*} \mathbf{p} \equiv \left( \begin{array}{c} p\left(x,t\right)\\ c\left(x,t\right) \end{array}\right),\,\,\,\, \quad \mathbb{L}\left(\mathbf{p}\right) \equiv \left( \begin{array}{c} -v_+\frac{\partial p}{\partial x} + D_+ \frac{\partial^2 p}{\partial x^2}\\ D\frac{\partial^2c}{\partial x^2} \end{array}\right)\end{equation*}

and

\begin{equation*} \mathbf{A} = \left( \begin{array}{cc} -k_{\mathrm{off},+} &amp; k_{\mathrm{on},+}\\ k_{\mathrm{off},+} &amp; -k_{\mathrm{on},+} \end{array}\right) \,.\end{equation*}

The co-kernel of **A** is spanned by the vector $\mathbf{v}^T = (1,1)$ and the kernel of **A** is spanned by the vector

\begin{equation*} \mathbf{u} = \frac{1}{k_{\mathrm{on},+} + k_{\mathrm{off},+}}\left( \begin{array}{c} k_{\mathrm{on},+}\\k_{\mathrm{off},+} \end{array}\right),\end{equation*}

so that $\mathbf{v}^T\mathbf{u} = 1$. Indeed **u** is the eigenvector corresponding to the zero eigenvalue of **A** and represents the stationary density of a Markov process formed by the motor switching rates.

Let $q = \mathbf{v}^T \mathbf{p} = p(x,t)+c(x,t)$ and $\mathbf{w} = \mathbf{p} - q\mathbf{u}$, so that *q* is proportional to the component of **p** in the co-kernel of **A** and **w** is in the orthogonal complement. Applying **v**^*T*^ to both sides of equation (8) gives

\begin{equation*} \frac{\partial q}{\partial t} = \mathbf{v}^T \mathbb{L}\left( \mathbf{w} + q\mathbf{u}\right)\,.\end{equation*}

Substituting $\mathbf{p} = \mathbf{w} + q\mathbf{u}$ into equation (8) and using equation (9) and the fact that $\mathbf{u}\in \mathrm{Ker}(\mathbf{A})$, we obtain

\begin{equation*} \frac{\partial }{\partial t} \mathbf{w} = \frac{1}{\varepsilon} \mathbf{A}\mathbf{w}+ \left(\mathbb{I}_2 - \mathbf{u}\mathbf{v}^T\right)\mathbb{L}\left(\mathbf{w} + q\mathbf{u}\right)\,,\end{equation*}

where $\mathbb{I}_2$ is the $2 \times 2$ identity matrix. We introduce the expansion

\begin{equation*} \mathbf{w} = \mathbf{w_0} + \varepsilon \mathbf{w_1}+ O\left(\varepsilon^2\right)\end{equation*}

and substitute it into equation (10). Collecting $O(\varepsilon^{-1})$ terms gives $\mathbf{A}\mathbf{w_0} = \mathbf{0} \Rightarrow \mathbf{w_0} = \mathbf{0}$, since we require $\mathbf{v}^T\mathbf{w_0} = \mathbf{0}$. In this case, higher order terms are not needed to extract a reasonable effective equation, so we do not compute higher order terms.

Substituting the leading order term for **w** ($\mathbf{w_0} = \mathbf{0}$) into equation (9) gives the effective advection-diffusion equation for $q(x,t)$, the total density of (+) moving motors at position *x* at time *t*:

\begin{equation*} \frac{\partial q}{\partial t} = \mathbf{v}^T\mathbb{L}\left(q\mathbf{u}\right) = -\mathscr{V}_+\frac{\partial q}{\partial x} + \mathscr{D}_+ \frac{\partial^2 q}{\partial x^2}\,,\end{equation*}

where

\begin{align*} {\mathscr{V}_+} &amp;\equiv \frac{k_{\mathrm{on},+}}{k_{\mathrm{on},+} + k_{\mathrm{off},+}} v_+ \quad {\mathrm{and}{}} \quad \nonumber\\ \mathscr{D}_+ &amp;\equiv \frac{k_{\mathrm{on},+}}{k_{\mathrm{on},+} + k_{\mathrm{off},+}} D_+ + \frac{k_{\mathrm{off},+}}{k_{\mathrm{on},+} + k_{\mathrm{off},+}} D\,.\end{align*}

We refer to $\mathscr{V}_+$ as the effective velocity of the (+) motors, and to $\mathscr{D}_+$ as the effective diffusivity of the (+) motors. After performing a similar approximation on the model of (–) moving motors, we obtain the following system of parabolic equations describing motor dynamics of (+) moving motors $q(x,t)$ and (–) moving motors $h(x,t)$:

\begin{align*} \begin{split} \frac{\partial q}{\partial t} &amp; = -\mathscr{V}_+\frac{\partial q}{\partial x} + \mathscr{D}_+ \frac{\partial^2 q}{\partial x^2}\\ \frac{\partial h}{\partial t} &amp; = \mathscr{D}_- \frac{\partial^2 h}{\partial x^2} \,, \end{split}\end{align*}

where

\begin{equation*} \mathscr{D}_- \equiv \frac{k_{\mathrm{on},-}}{k_{\mathrm{on},-} + k_{\mathrm{off},-}} D_- + \frac{k_{\mathrm{off},-}}{k_{\mathrm{on},-} + k_{\mathrm{off},-}} D\,.\end{equation*}

These are coupled with the boundary conditions:

\begin{align*} \begin{split} &amp;q\left(L_-\right) = 1 \quad \quad \quad \quad\quad\quad h\left(L_-\right) + \frac{\partial h}{\partial x}\Big|_{x = L_-} = 1\\ &amp;\mathscr{V}_+q\left(L_+\right) - \mathscr{D}_+ \frac{\partial q}{\partial x} \Big|_{x = L_+} = 1 \quad \quad h\left(L_+\right) = 1. \end{split}\end{align*}

We note that the Robin conditions arise from the fast switching between Neumann and Dirichlet conditions [35].

### 4.2. Adiabatic approximation and analysis

We make the further assumption that effective motor velocities are greater than the growth rates of the microtubules, to ensure that motors can reach the end of the microtubules and induce motor-dependent shrinking before the microtubule reaches its basal equilibrium length. Implementing this approximation will allow us to investigate and make predictions regarding those regimes where such assumptions hold. Acquiring such data about an application system is challenging, however this is exactly where mathematical modeling is especially useful. We can leverage it to make predictions about what could happen in the particular parameter regimes that correspond to the approximation assumptions here.

We assume the motor dynamics reach equilibrium before studying microtubule length dynamics. The equilibrium solutions are readily obtainable from equations (12) and (13), yielding

\begin{align*} q\left(x\right) &amp; = \left(1 + \frac{1}{\mathscr{V}_+}\right)\mathrm{exp}\left(\frac{\mathscr{V}_+}{\mathscr{D}_+}\left(x - L_-\right)\right)- \frac{1}{\mathscr{V}_+}\\ h\left(x\right) &amp; = 1 \,.\end{align*}

Substituting these into equations (7) gives

\begin{align*} \begin{split} \frac{\mathrm{d}L_+}{\mathrm{d}t} &amp; = \alpha_+ - \beta_+\left(L_+-L_-\right) - \gamma_+\nonumber\\ &amp;\quad\times\left( \left(1 + \frac{1}{\mathscr{V}_+}\right)\mathrm{exp}\left(\frac{\mathscr{V}_+}{\mathscr{D}_+}\left(L_+ - L_-\right)\right)- \frac{1}{\mathscr{V}_+}\right)\\ \frac{\mathrm{d}L_-}{\mathrm{d}t} &amp; = -\alpha_- + \beta_-\left(L_+-L_-\right) + \gamma_- \,. \end{split}\end{align*}

Defining $L \equiv L_+ - L_-$ yields the 1D flow for the length of the microtubule:

\begin{align*} \frac{\mathrm{d}L}{\mathrm{d}t} &amp;= \left(\alpha_+ + \alpha_-\right) - \left(\beta_+ + \beta_-\right)L - \gamma_+\nonumber\\ &amp;\quad\times\left( \left(1 + \frac{1}{\mathscr{V}_+}\right)\mathrm{exp}\left(\frac{\mathscr{V}_+}{\mathscr{D}_+}L\right)- \frac{1}{\mathscr{V}_+}\right) - \gamma_- \,.\end{align*}

Equation (15) yields a unique equilibrium microtubule length provided $\alpha_+ + \alpha_- > \gamma_+ + \gamma_-.$ This is straightforward to see by rewriting equation (15) as

\begin{align*} \frac{\mathrm{d}L}{\mathrm{d}t} = f\left(L\right) - g\left(L\right)\,,\end{align*}

where

\begin{align*} f\left(L\right) &amp;= \left(\alpha_+ + \alpha_-\right) - \left(\beta_+ + \beta_-\right)L \quad \quad g\left(L\right) = \gamma_+\nonumber\\ &amp;\quad\times\left( \left(1 + \frac{1}{\mathscr{V}_+}\right)\mathrm{exp}\left(\frac{\mathscr{V}_+}{\mathscr{D}_+}L\right)- \frac{1}{\mathscr{V}_+}\right) + \gamma_-.\end{align*}

**Figure 2.**
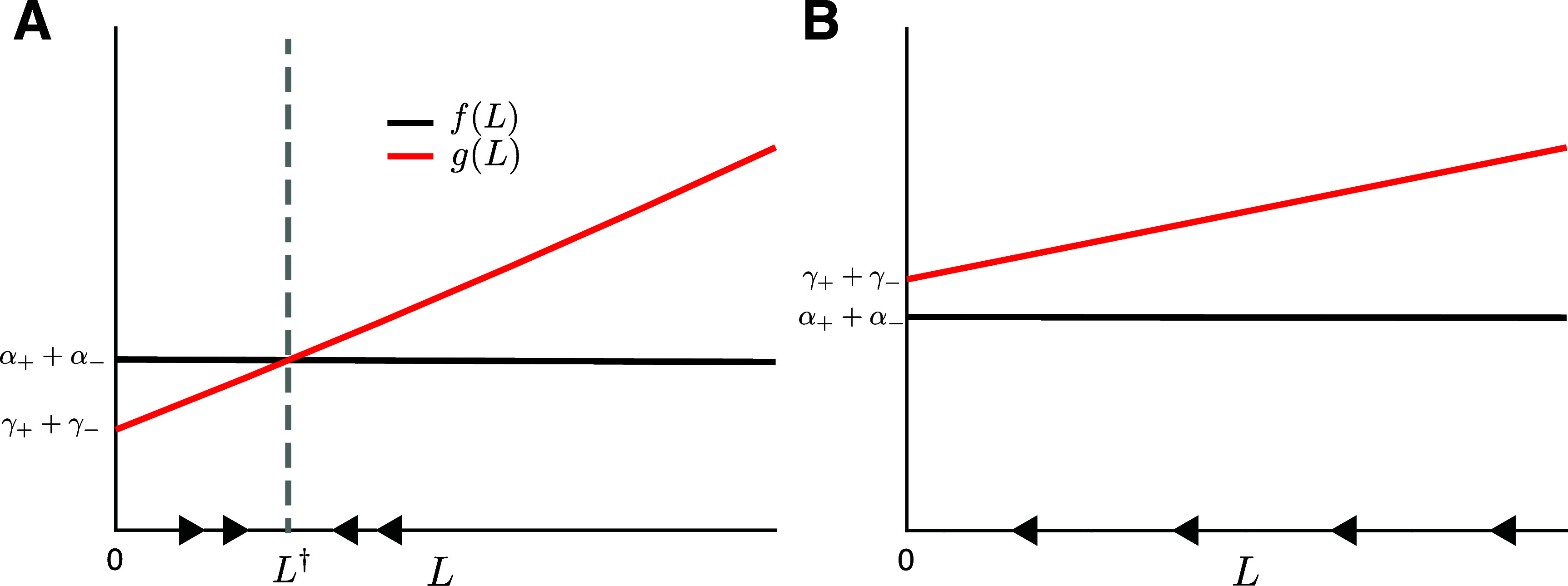
Dynamics of equation (15) in different parameter regimes. (A) If $\alpha_+ + \alpha_- > \gamma_+ + \gamma_-$, the model predicts the existence of a unique equilibrium microtubule length, $L^{\dagger}$; (B) If $\alpha_+ + \alpha_- < \gamma_+ + \gamma_-$, then the microtubule shrinks.

Since $f^{^{\prime}}(L) < 0$ and $g^{^{\prime}}(L) > 0$, $\forall L$, if $\alpha_+ + \alpha_- > \gamma_+ + \gamma_-$ (i.e. if $f(0) > g(0)$), then the Intermediate Value Theorem guarantees that *f* and *g* intersect for some *L* > 0. The intersection point is the unique microtubule length, $L^\dagger$ (see figure 2).

**Figure 3.**
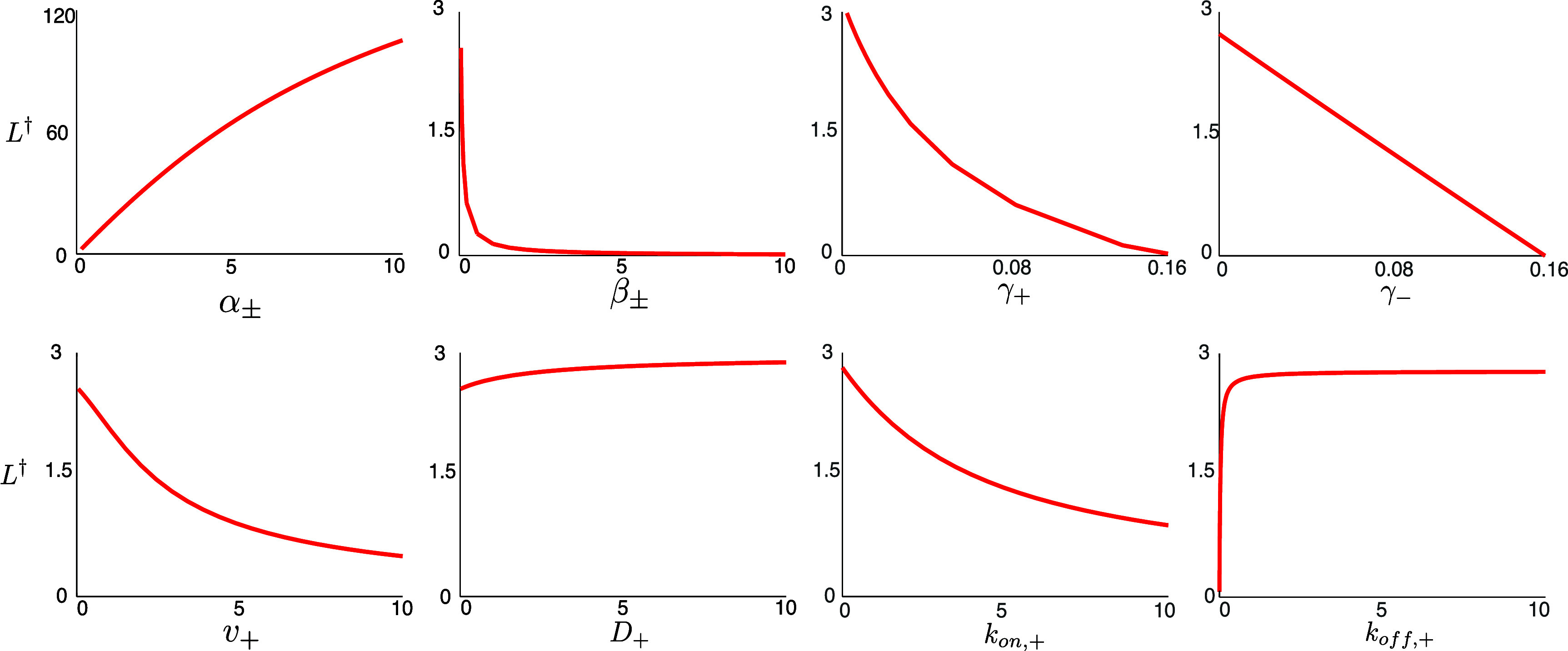
Dependence of $L^{\dagger}$ upon various parameters in the reduced model in equation (15).

An interesting aspect of the reduced model is the prediction that the basal microtubule shrinking rates, $\beta_{\pm}$, do not affect the existence of an equilibrium length but only its value (see figure 3). Only the basal growth rates of the microtubules and the motor-dependent shrinking rates are responsible for establishing whether a microtubule reaches an equilibrium length or shrinks to zero.

*Treadmilling.* A generic equilibrium resulting from equation (15) results in a treadmilling equilibrium, meaning $\frac{\mathrm{d}L}{\mathrm{d}t} = 0$ but $\frac{\mathrm{d}L_+}{\mathrm{d}t}, \frac{\mathrm{d}L_-}{\mathrm{d}t} \neq 0$. This is another key prediction of the model: equilibrium microtubule lengths are effectively *only* maintained *dynamically* with growing and shrinking tips rather than fixed tips.

Equilibrium tip positions can be obtained in the reduced model, (15), but they occur in very constrained regimes of parameter space. To have an equilibrium point $(L_+^{\dagger}, L_-^{\dagger})$ of equations (14), we must have $\frac{\mathrm{d}L_+}{\mathrm{d}t} = 0$ and $\frac{\mathrm{d}L_-}{\mathrm{d}t} = 0$. Consider the nullclines of the system obtained by setting each equation individually equal to zero. The nullclines $n_+(L_+,L_-)$ and $n_-(L_+,L_-)$ satisfy

\begin{equation*} n_+\left(L_+,L_-\right) = 0 \quad \quad n_-\left(L_+,L_-\right) = 0\,,\end{equation*}

where

\begin{align*} n_-\left(L_+,L_-\right) &amp; = L_+ - L_- - \frac{\alpha_- - \gamma_-}{\beta_-}\\ n_+\left(L_+,L_-\right) &amp; = L_+ - L_- - \left(\frac{\mathscr{D}_+}{\beta_+\mathscr{V}_+}\right)\nonumber\\ &amp;\quad\times\left(-\beta_+\Omega\left(\frac{\frac{\mathscr{V}_+}{\mathscr{D}_+}\gamma_+\left(1 + \frac{1}{\mathscr{V}_+}\right)\mathrm{exp}\left(\frac{\mathscr{V}_+}{\mathscr{D}_+\beta_+}\left(\alpha_+ + \frac{\gamma_+}{\mathscr{V}_+}\right)\right)}{\beta_+}\right)+\frac{\alpha_+\mathscr{V}_+ + \gamma_+}{\mathscr{D}_+}\right),\end{align*}

where $\Omega(x)$ is the omega function or the Lambert-W function. Both nullclines are lines with unit slope in phase space, so they can only intersect when

\begin{align*} \frac{\alpha_- - \gamma_-}{\beta_-} &amp;= \left(\frac{\mathscr{D}_+}{\beta_+\mathscr{V}_+}\right)\left(-\beta_+\Omega\left(\frac{\frac{\mathscr{V}_+}{\mathscr{D}_+}\gamma_+\left(1 + \frac{1}{\mathscr{V}_+}\right)\mathrm{exp}\left(\frac{\mathscr{V}_+}{\mathscr{D}_+\beta_+}\left(\alpha_+ + \frac{\gamma_+}{\mathscr{V}_+}\right)\right)}{\beta_+}\right)+\frac{\alpha_+\mathscr{V}_+ + \gamma_+}{\mathscr{D}_+}\right).\end{align*}

In words, equation (16) says that equilibrium microtubule length with fixed ends can be attained only if motor dynamics in the (+) end and (-) end are roughly equal. This in general will not be the case, simply due to major biophysical differences. Thus, we consider the case of fixed endpoints as a special constrained case that will be rare in biological settings.

Our model is thus amenable to analysis and makes concrete predictions about microtubule length at equilibrium. However, the assumptions about the parameters justifying the approximations do not cover the most general regimes. We thus next perform simulations based on parameters drawn from experimental data.

## 5. Full model simulations

We now return to the full model formulation in equations (1) and (7). We are interested in modeling specific microtubule-depolymerizing motors that have been identified in experiments and whose dynamics has been at least partially quantified. In particular, we consider the effect of (+)-end motor kinesin-8 and (–)-end motor kinesin-13, whose roles as microtubule-depolymerizing motors are described in section 2. Therefore, the fast-switching limit in section 4.1 and the assumption of rapid motor equilibration in section 4.2 may not hold, and we thus must consider efficient numerical simulations of the full coupled model of motor and MT behavior.

### 5.1. Model implementation and parametrization

In equations (1) and (7), the spatial domain of the PDEs (i.e. the length of the MT) changes through time. We therefore consider efficient numerical methods for moving the domain boundaries and adjusting the computation mesh. We implement equations (1) with boundary conditions (5)–(7) in FlexPDE, which supports moving computation meshes in time-dependent problems [36]. This means that we distribute the computation mesh smoothly within the moving domain boundary by using a surrogate coordinate for the spatial dimension *x* and by using the diffusion equations for distributing the positions smoothly in the interior.

To parametrize the model, we use experimental measurements for the dynamics of motors and MT ends whenever possible. Table 1 outlines the parameters used to evaluate the model. In the kinetic parameters characterizing motor dynamics, we note that the (+)-end motor (modeling kinesin-8) has a slow diffusivity along microtubules, while the (–)-end motor (kinesin-13) does not use directed motion and instead has a high diffusion coefficient for movement along microtubules. The binding/association rates are typically measured in the literature as a function of the concentration of motors, which is not consistent with our model framework. We therefore set these parameters to a baseline value in this study. We note that several known parameters are not identical for (+)- and (–)-end motors.

**Table 1.** Parameters used for the full model formulation, informed from *in vitro* and *in vivo* experiments.

Motor parameters	Notation	Plus-end (Source)	Minus-end (Source)
Motor speed on MTs	$v_{+,-}$	0.05 *µ*m s^−1^ [9]	0 [25, 31]
Motor diffusion on MTs	$D_{+,-}$	0.01 *µ*m^2^ s^−1^ (this study)	0.38 *µ*m^2^ s^−1^ [31]
Motor diffusion off MTs	*D*	4 *µ*m^2^ s^−1^ [40]	4 *µ*m^2^ s^−1^ [40]
Binding rate to MTs	$k_{\mathrm{on},+/-}$	1 (this study)	1 (this study)
Unbinding rate from MTs	$k_{\mathrm{off},+/-}$	0.25 s^−1^ [41]	1.2 s^−1^ [31]—$3\,\mathrm{s}^{-1}$ [42]

MT parameters	Notation	Plus-end (Source)	Minus-end (Source)

Intrinsic MT growth speed	$\alpha_{+,-}$	0.15 *µ*m s^−1^ [37]	0.02 *µ*m s^−1^ [37]
Length-dependent shrinking rate	$\beta_{+,-}$	$0.05\,\mathrm{s}^{-1}$, varies in this study	$0.0006\,\mathrm{s}^{-1}$, varies in this study
Motor-driven shrinking rate	*γ*	0.01, varies in this study	0.01, varies in this study

To inform the parameters in boundary conditions (7) for the impact of the motors on the MT (+) and (–) ends, we use emergent MT length measurements from *in vivo* sensory neurons of the *Drosophila* larva [37]. The maximum speeds of growth at the (+) and (–) ends of MTs in this system are given by $v_g^{+,\mathrm{max}} = 9\,\mu$m min^−1^ and $v_g^{-,\mathrm{max}} = 1.125\,\mu$m min^−1^. If the microtubule polymerization at the ends is driven entirely by intrinsic growth, equations (7) would become

\begin{equation*}\frac{\mathrm{d}L}{\mathrm{d}t} = \alpha\,,\end{equation*}

and thus parameters $\alpha_{+/-}$ can be set to the maximal MT growth speeds. Since we use a timescale of seconds in our model simulations, we approximate these parameters to be $\alpha_+ = v_g^{+,\mathrm{max}} = 0.15\,\mu$m s^−1^ and $\alpha_- = v_g^{-,\mathrm{max}} = 0.02\,\mu$m s^−1^.

Length- or age-dependent catastrophe of MTs is a documented phenomenon of MT dynamics. Experiments have shown that catastrophe is suppressed in short newly-polymerizing microtubules [23], and the initial simple models of stochastic changes in GTP cap length leading to catastrophe could not account for the increase in catastrophe frequency as microtubules grow. However, there are various mechanisms that could explain length-dependent shrinking. The accumulation of variable protofilament lengths as microtubules elongate [26] and the accumulation of microtubule-depolymerizing motors at growing MT ends (antenna effect) [9, 25] are likely to both contribute to the observed length- or age-dependent catastrophe. In recent work, we showed that tuning the length-dependent catastrophe rate can have a significant impact on the resulting MT length distributions in a realistic stochastic model [38, 39].

Therefore, setting parameters $\beta_{+,-}$ and $\gamma_{+,-}$ in equations (7) is a difficult task. To inform rough starting values for parameters $\beta_{+,-}$, we again consider measurements from *in vivo* sensory neurons of the Drosophila larva. The average speeds of growth at the (+) and (–) ends of *Drosophila* sensory neurons are $v_g^{+,\mathrm{avg}} = 6\,\mu$m min^−1^ and $v_g^{-,\mathrm{avg}} = 0.75\,\mu$m min^−1^. If we make the assumption that these average growth speeds reflect the intrinsic MT growth and the length-dependent shrinking due to ragged protofilament ends only [26], equations (7) become

\begin{equation*}\frac{\mathrm{d}L}{\mathrm{d}t} = \alpha - \beta L\,.\end{equation*}

We set a characteristic MT length $\bar{L} = 10\,\mu$m, which is a relevant length scale for MTs in these neurons. Estimating that $\frac{\mathrm{d}L}{\mathrm{d}t} \approx v_g^{\mathrm{avg}}$, and using the choices of $\alpha_{+/-}$ from the maximal MT growth speeds, we estimate that $\beta_+ = \frac{3\,\mu\mathrm{m\,min^{-1}}}{10\,\mu\mathrm{m}} = 0.05\,\mathrm{s}^{-1}$ and $\beta_- = \frac{0.375\,\mu \mathrm{m\,min^{-1}}}{10\,\mu \mathrm{m}} = 0.0006\,\mathrm{s}^{-1}$. We re-iterate that these are simply starting values for our numerical simulations, and that we are interested in how changes in these parameters impact the model MT length. In addition, since we are also interested in the impact of the depolymerizing motor populations on MT length, we expect that parameters $\gamma_{+,-}$ from equations (7) may also need to be varied. The initial value for these parameters is in the range investigated in [38].

To identify distinct MT behaviors in different parameter regimes, we next perform parameter sweeps with particular parameter directions informed by the results of the reduced model (figure 3) and numerical simulations of the full model. Our baseline parameter set is as discussed above.

### 5.2. Model behavior

We now assess the behavior of the predicted motor densities and MT length based on the full model in section 3 using numerical implementation in FlexPDE [36] and the parameters in table 1. We observe several qualitative behaviors that emerge for the predicted MT length and the MT end dynamics.

**Figure 4.**
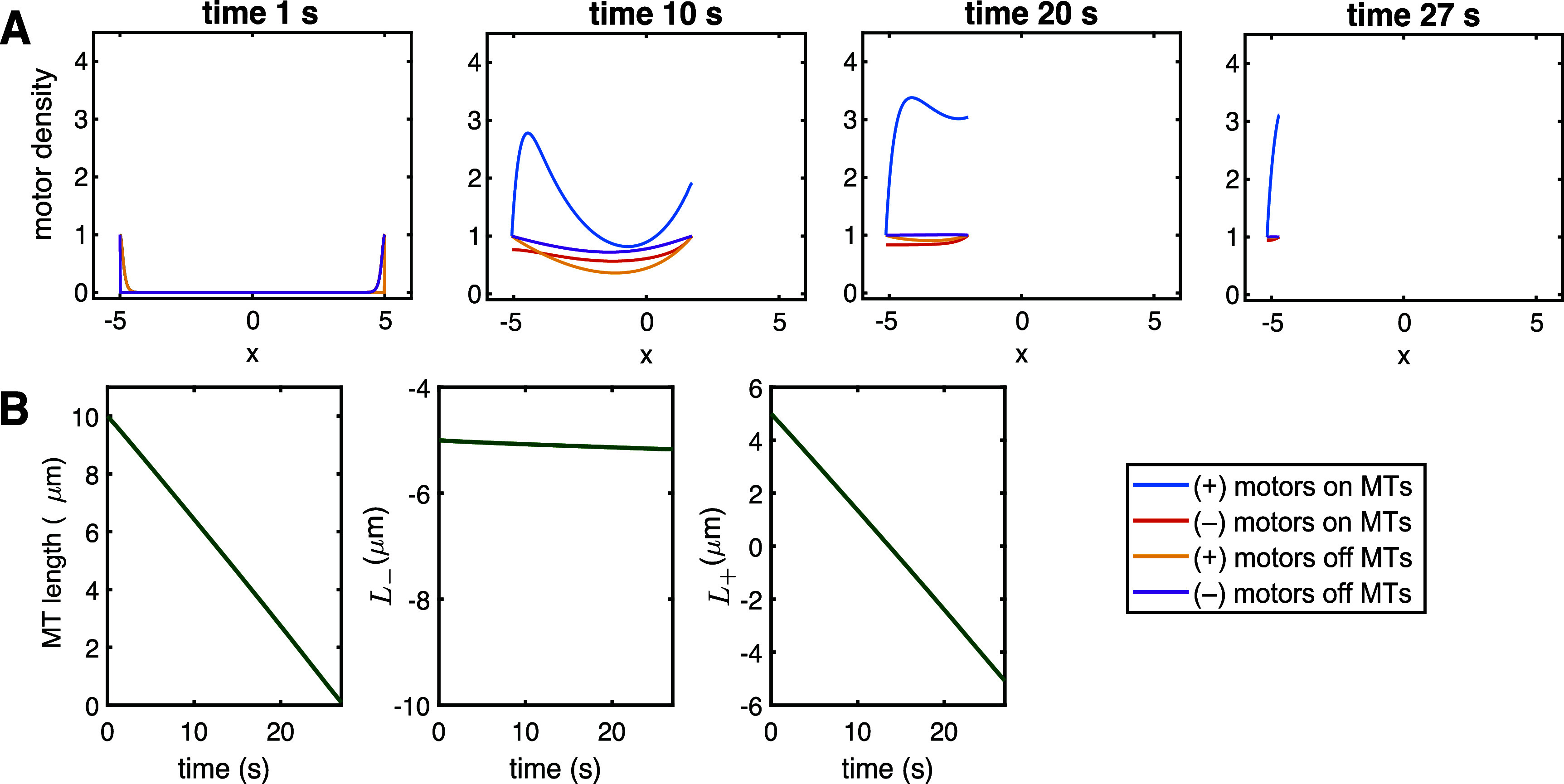
(a) Full model predictions of motor population densities for $\beta_+ = 0.05\,\mathrm{s}^{-1}$, $\beta_- = 0.0006\,\mathrm{s}^{-1}$ and $\gamma_+ = \gamma_- = 0.01$ (disassembly). (b) Corresponding evolution of the underlying MT length and the predicted dynamics at the (+) and (–) ends.

*Disassembly.* The baseline parameters discussed and derived in section 5.1 lead to fast disassembly of a MT starting at the characteristic length $\bar{L} = 10\,\mu$m. Figure 4(a) shows the evolution of each motor population density along the shrinking MT, while figure 4(b) shows the linear decrease in MT length and the dynamics at each MT end. These baseline parameters predict slow, sustained growth at the (–) end of MTs (as observed in [37]) and demonstrate rapid disassembly at the (+) end of the model MT (see Supplementary Video 1). Since the behavior of the (+)- and (–)-end motors is not symmetric (i.e. it is characterized by different parameters), this leads to the emergence of different sizes of the densities of motor populations.

**Figure 5.**
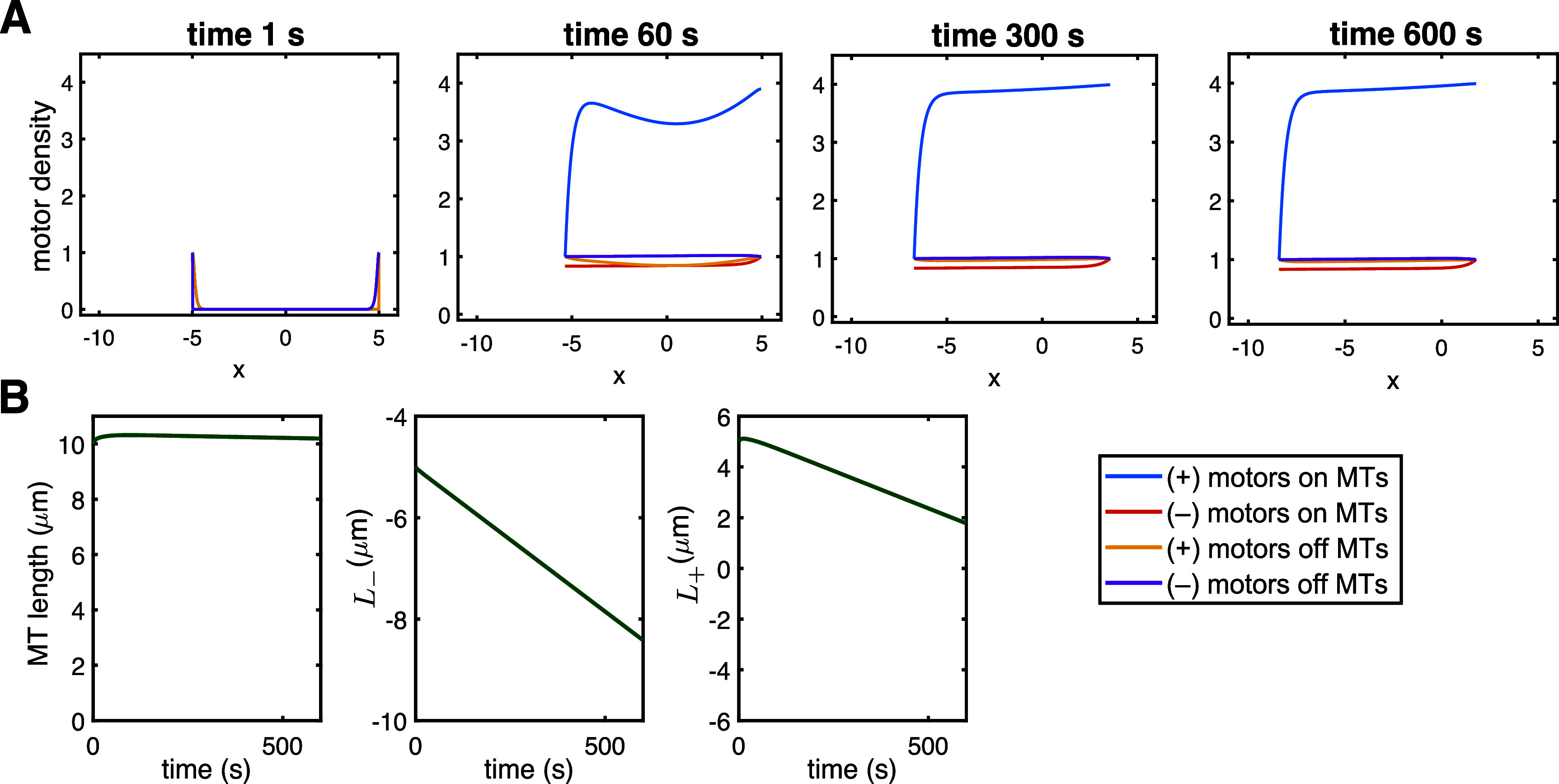
(A) Full model predictions of motor population densities for $\beta_+ = 0.0116\,\mathrm{s}^{-1}$, $\beta_- = 0.0006\,\mathrm{s}^{-1}$ and $\gamma_+ = \gamma_- = 0.01$ (treadmilling). (B) Corresponding evolution of the underlying MT length and the predicted dynamics at the (+) and (–) ends.

*Treadmilling.* In figure 5, we explore whether the full model can exhibit MT treadmilling, where one end of the filament grows while the other end shrinks. This MT behavior has been documented and reviewed in the experimental literature [43]. By tuning the length-dependent disassembly rate at the (+) end (parameter $\beta_+$), we show that MT treadmilling is achieved and maintained in the full model. Figure 5 shows that the MT length stays constant at the characteristic length $\bar{L} = 10\,\mu$m, and that all motor populations achieve steady-state within minutes and move along the treadmilling MT. We run this simulation for 10 min to illustrate that the MT treadmilling movement predicted by the model is sustained through time (see Supplementary Video 2).

**Figure 6.**
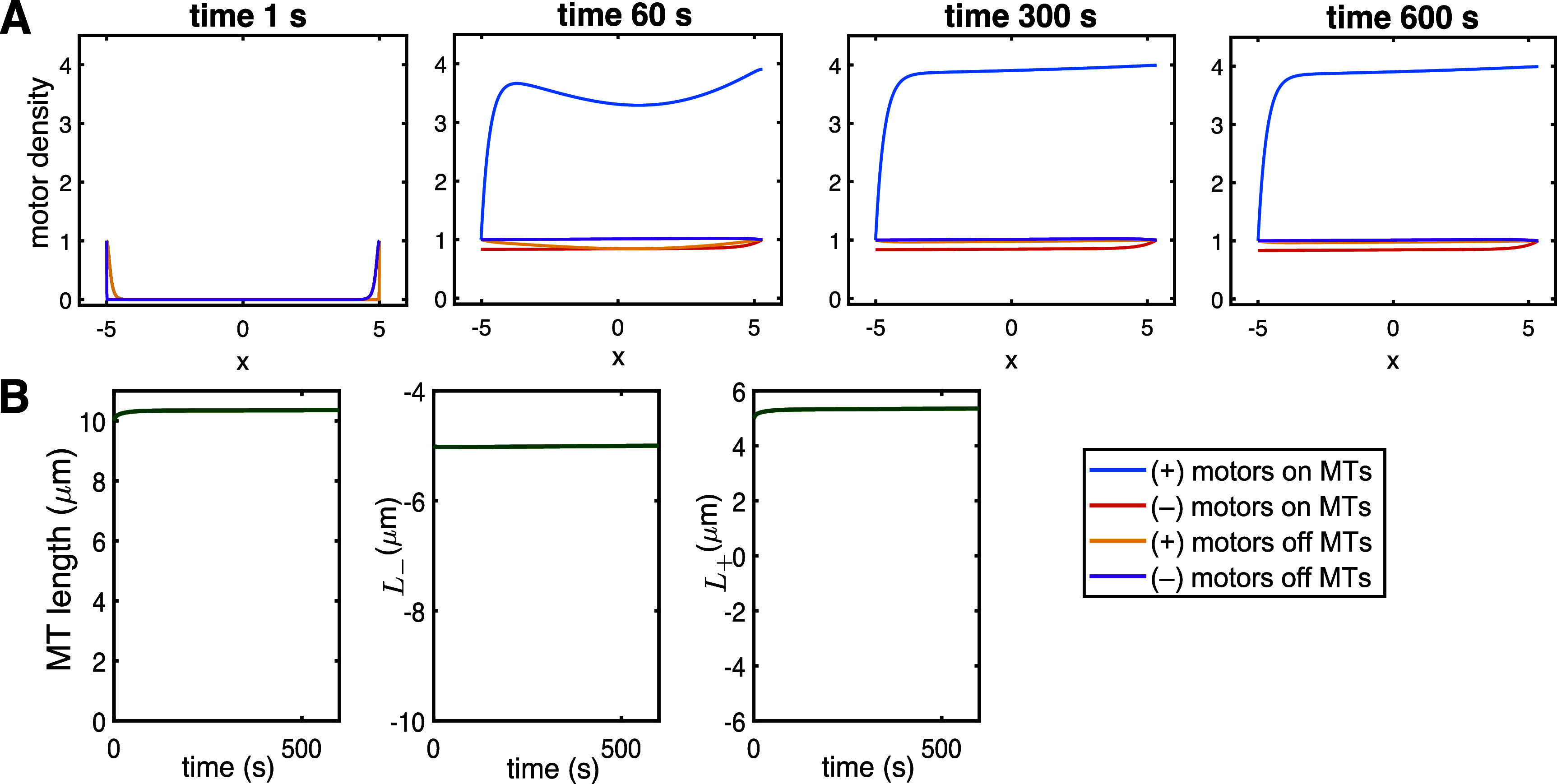
(A) Full model predictions of motor population densities for $\beta_+ = 0.011\,\mathrm{s}^{-1}$, $\beta_- = 0.00117\,\mathrm{s}^{-1}$ and $\gamma_+ = \gamma_- = 0.01$ (equilibrium). (B) Corresponding evolution of the underlying MT length and the predicted dynamics at the (+) and (–) ends.

*Fixed MT ends.* figure 6 shows that the full model can also illustrate equilibrium behavior at both MT ends. This is achieved by keeping parameters the same as in the treadmilling case, except that we are further tuning the length-dependent disassembly at the (–) end (parameter $\beta_-$). This leads to fixed MT ends and maintaining the equilibrium length of $\bar{L} = 10\,\mu$m (see Supplementary Video 3). We note that this behavior occurs in very constrained regions of the parameter space, where significant tuning of the intrinsic length-dependent disassembly mechanism at both ends is required. Indeed, this is consistent with what the reduced model suggested in section 4.2.

**Figure 7.**
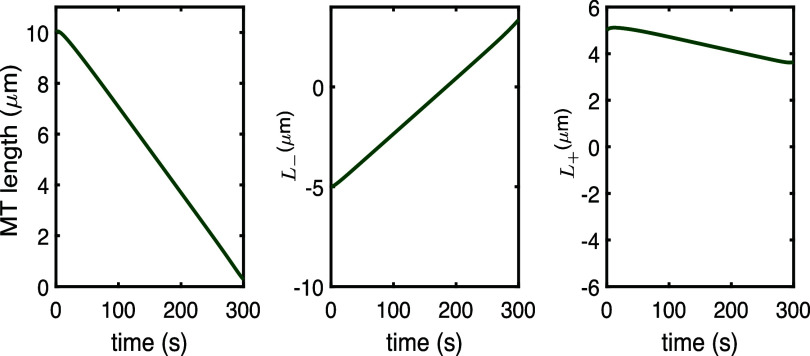
Full model predictions of the evolution of the MT length and the dynamics at the (+) and (–) ends for $\beta_+ = 0.0116\,\mathrm{s}^{-1}$, $\beta_- = 0.0006\,\mathrm{s}^{-1}$, $\gamma_+ = 0.01$, and $\gamma_- = 0.05$ (disassembly due to Patronin RNAi perturbation).

*Disassembly through Patronin RNAi perturbations.* The full model also allows for testing changes that correspond to experimental perturbations. For example, Patronin is a protein that acts as a brake on (–)-end microtubule depolymerization by kinesin-13. This protein specifically binds to and protects the (–) ends of MTs, preventing them from being disassembled by kinesin-13. Assuming that our full model includes this protective impact of Patronin on (–) ends (through the choice of the $\gamma_-$ parameter), we can model Patronin RNAi experiments as in [30] by increasing $\gamma_-$. This change means that our model predicts full microtubule disassembly (figure 7), consistent with the complete MT disappearance observed in experiments [30].

The full model can thus generate realistic MT end behaviors given assumptions about different regulation of microtubule end dynamics and length-dependent disassembly. This model can be easily updated with additional biological insights and relevant parameters to provide predictions for cytoskeleton behavior in specific applications. Unlike the reduced model in section 4.2, exploring the behavior of this more complex model and its parameter space requires growing domain simulations, which impose a higher computational cost.

## 6. Discussion

In this paper, we proposed a model for understanding non-centrosomal microtubule dynamics that accounts for microtubule tip dynamics at both the (+) and (-) ends. Our model incorporates motor-based microtubule depolymerization and allows us to assess the impact of motor dynamics on microtubule growth and shrinking dynamics. Importantly, some parameters that inform our model of microtubule end dynamics are based on *in vivo* measurements from neuronal microtubules. Exploring the parameter space of our model and the emergent microtubule behaviors allows for predictions of cytoskeleton dynamics in living neurons. We emphasize that such parameter space exploration is important for our biophysical understanding of microtubule dynamics in living cells, since obtaining experimental data about these processes is extremely challenging. Moreover, understanding microtubule dynamics and their polarized organization within cells can provide insight into how these filaments mediate the transport of protein cargo and thereby contribute to the sorting of cellular components. In principle, the full model can classify MT behaviors across a wide range of parameter values, but the computational cost of such a classification is substantial. To address this, we derived a reduced model which greatly facilitates understanding MT behavior in distinct parameter regimes and provides elegant analytic results.

The model simulations presented here show that tuning mechanisms that influence the disassembly behavior of microtubules can lead to maintenance of an equilibrium length. In neurons, this is very important, since these cells must last a lifetime and must maintain a relatively uniform coverage of microtubules throughout the axon and dendrite [44]. In particular, stable microtubules and microtubule coverage of these neurites allows for transport of key proteins throughout neurons. In our model, different equilibrium lengths can be obtained with different choices of free parameters. This reflects what is known about the variability of microtubule lengths; filaments are known to be tuned spatially and to vary in axons and dendrites of neurons and in different organisms [44]. There are also many parameter regimes of the model where microtubules undergo shrinking. We can connect this with the neurons’ ability to respond to stimuli. For example, in *Drosophila* fruit fly neurons, microtubules in a selected dendrite have been shown to reverse their polarity following axon injury, which requires that they first undergo complete disassembly [45]. Finally, we also observe microtubule treadmilling in our model. Such microtubule sliding relies on motor proteins and cross-linkers and has been found to be key for axonal growth, neurite formation, and for maintenance of microtubule polarity in neurons [46, 47].

Furthermore, the analytical and computational results presented here offer immediate biophysical insights into cellular structures that rely on simultaneous, two-ended microtubule regulation, such as the mitotic spindle. During metaphase, kinetochore microtubules exhibit a continuous ‘poleward flux’, characterized by tubulin addition at the kinetochore (+) end and removal at the spindle pole (−) end [48, 49], maintaining a relatively constant filament length while generating critical mechanical tension across sister chromatids [37, 50–53]. Our reduced multiscale model demonstrates that such treadmilling behavior is not an anomalous state requiring complex external orchestration, but rather a robust, emergent property of the system whenever equilibrium is achieved via motor regulation at both ends.

Numerical simulations of our model, which includes a growing domain, allow for identifying qualitatively different behaviors of microtubules. This, in turn, facilitates evaluation of the contribution of distinct mechanisms (i.e, motor and non-motor driven) to the observed length-dependent disassembly of microtubules. While we focus on kinesin-8 and kinesin-13 depolymerizing motors here, the model can be extended to other motor proteins that have been found to have an impact on dynamic instability of microtubules *in vivo* and *in vitro*. For example, dynein has been found to regulate (+)-end stability and control microtubule length in budding yeast [54] and in a reconstituted cortical system [55]. In specific model parameter regimes, we can invoke a quasi-steady state and adiabatic approximation, which allows us to derive a tractable mathematical model and thus directly determine the role that various biophysical parameters play in affecting microtubule length.

Notably, we did not investigate the dynamics of our model in the fast-switching limit but without the adiabatic approximation. In this regime, we could, in principle, find an explicit solution for (+) and (–) motor densities in terms of the corresponding causal Green’s functions of the parabolic operators governing motor dynamics. This would be interesting mathematically and biophysically, as the model would make predictions about microtubule length dynamics to be verified experimentally, and it involves Green’s functions with a domain that changes in time. In particular, it would be interesting to perform stability analysis on this reduced model.

There are a number of other avenues we plan to explore in the future. First, we would like to develop a more biophysically-detailed model that captures motor-motor interactions and to investigate how these interactions impact microtubule length dynamics. One possible implementation is to allow the motors to evolve according to a totally asymmetric simple exclusion process [56–58], thereby incorporating exclusion effects between motors. We could also investigate motor dynamics on an ensemble of microtubules, each undergoing motor-dependent shrinking, and to observe the resulting equilibriumdistribution of microtubule lengths. This would provide particularly interesting theoretical predictions, since distributions of microtubule length are challenging to obtain experimentally in living cells such as neurons. Finally, we could relate ensemble microtubule length distribution with the macroscopic neuronal length, seeking to answer the question: how do subcellular filamentous structures scale in size with neuronal length? This could be achieved, for example, by linking microtubule length dynamics and motor dynamics with axonal length sensing [33, 59, 60].

## Data Availability

All data that support the findings of this study are included within the article (and any supplementary files). Supplementary Video 1 available at https://doi.org/10.1088/1478-3975/ae600a/data1. Supplementary Video 2 available at https://doi.org/10.1088/1478-3975/ae600a/data2. Supplementary Video 3 available at https://doi.org/10.1088/1478-3975/ae600a/data3.
